# GDNF synthesis, signaling, and retrograde transport in motor neurons

**DOI:** 10.1007/s00441-020-03287-6

**Published:** 2020-09-08

**Authors:** Alberto F. Cintrón-Colón, Gabriel Almeida-Alves, Alicia M. Boynton, John M. Spitsbergen

**Affiliations:** Department of Biological Sciences, Western Michigan University, Kalamazoo, MI 49008 USA

**Keywords:** GDNF signaling, GDNF, Motor neuron

## Abstract

Glial cell line–derived neurotrophic factor (GDNF) is a 134 amino acid protein belonging in the GDNF family ligands (GFLs). GDNF was originally isolated from rat glial cell lines and identified as a neurotrophic factor with the ability to promote dopamine uptake within midbrain dopaminergic neurons. Since its discovery, the potential neuroprotective effects of GDNF have been researched extensively, and the effect of GDNF on motor neurons will be discussed herein. Similar to other members of the TGF-β superfamily, GDNF is first synthesized as a precursor protein (pro-GDNF). After a series of protein cleavage and processing, the 211 amino acid pro-GDNF is finally converted into the active and mature form of GDNF. GDNF has the ability to trigger receptor tyrosine kinase RET phosphorylation, whose downstream effects have been found to promote neuronal health and survival. The binding of GDNF to its receptors triggers several intracellular signaling pathways which play roles in promoting the development, survival, and maintenance of neuron-neuron and neuron-target tissue interactions. The synthesis and regulation of GDNF have been shown to be altered in many diseases, aging, exercise, and addiction. The neuroprotective effects of GDNF may be used to develop treatments and therapies to ameliorate neurodegenerative diseases such as amyotrophic lateral sclerosis (ALS). In this review, we provide a detailed discussion of the general roles of GDNF and its production, delivery, secretion, and neuroprotective effects on motor neurons within the mammalian neuromuscular system.

## Introduction

Neurotrophic factors (NTFs) are a class of proteins that promote neuronal survival, control cell proliferation and differentiation, are required for axonal and dendritic elaborations, and regulate synaptic plasticity (Yan et al. 1995; Henderson et al. 1994; Zhu et al. 2008). NTFs include nerve growth factor (NGF), brain-derived neurotrophic factor (BDNF), neurotrophin-3 (NT-3), neurotrophin-4 (NT-4) belonging to the neurotrophins family, ciliary neurotrophic factor belonging to the CNTF family, and our neurotrophic factor of interest, glial cell line–derived neurotrophic factor (GDNF) belonging to the GDNF family ligands (GFLs) (Cobianchi et al. 2017; Oppenheim et al. 1995). One of the main functions of neurotrophic factors is to act as neurocytokines and, upon synthesis and secretion, to facilitate communication between neurons and their target tissues (Morcuende et al. 2013).

GFLs have four members: GDNF, neurturin, persephin, and artemin. The members of this family have low amino acid sequence homology, but all function as homodimers for the receptor tyrosine kinase rearranged during transfection (RET) activation. GDNF was originally isolated from cultured B49 rat glial cells and found to enhance the survival and differentiation of dopaminergic neurons in primary cultures by promoting dopamine uptake (Lin et al. 1993, 1994).

The GDNF sequence places the trophic factor in the cysteine knot growth factor superfamily. Although GDNF has a low overall protein sequence similarity to TGF-β2, the pattern of cysteine residues makes it a distant member of the TGF-β family (Lin et al. 1993). GDNF has two finger-like structures that make contact with the GFRα receptor. The location where N-linked glycosylation takes place is found close to the tip of one of the finger-like structures (Eigenbrot and Gerber 1997; Parkash et al. 2008; Silvian et al. 2006). The C-terminal of the mature GDNF has been established as highly important for its binding property to GFRα1 receptor and activation of RET (Eketjäll et al. 1999; Parkash et al. 2008). In the C-terminal of mature GDNF, we can find cysteines Cys131 and Cys133. These cysteines participate in the formation of a ring structure by linking with Cys68 and Cys72 (Oh-hashi et al. 2009).

Shortly after the discovery of GDNF, multiple researchers began identifying which cell types in the mammalian body contained and produced GDNF (Springer et al. 1995). GDNF protein is widely distributed throughout both the central and peripheral nervous systems. Synthesis and secretion of GDNF occur in many cell types such as glial cells like astrocytes, oligodendrocytes, and Schwann cells; motor neurons (MNs); and skeletal muscle. GDNF signaling has also been found within the normal growth and morphogenesis of the ureteric bud in developing the kidneys and in Sertoli cells in the testis (Henderson et al. 1994; Yan et al. 1995; Yamamoto et al. 1996; Meng et al. 2000; Costantini 2010).

GDNF has the most prominent effects on enteric, sympathetic, and dopamine neurons, but it has been identified as a potent neurotrophic factor for regulating MN survival in the peripheral nervous system (Henderson et al. 1994). GDNF prevents apoptosis of MNs during development in vivo (Oppenheim et al. 1995), decreases the loss of MNs in animal models of motor neuropathy and degeneration, rescues MNs from axotomy-induced cell death, and protects MNs from chronic degeneration (Li et al. 1995; Ruven et al. 2018; Trupp et al. 1995; Sariola and Saarma 2003; Airaksinen and Saarma 2002).

## GDNF synthesis, secretion, and internalization

GDNF is a secretory protein and is first formed as a precursor of 211 amino acids in mammalian cells. The pre-sequence leads the protein to the endoplasmic reticulum for secretion. As secretion takes place, the protein folds with sulfide (S–S) bonds, dimerizes, and later modified by N-linked glycosylation, and finally undergoes proteolytic processing into its mature form of 134 amino acids. This cleavage is due to a proteolytic consensus sequence located in exon II (Lin et al. 1993; Lin et al. 1994; Piccinini et al. 2013).

Proteases that play a role in the cleavage of pro-GDNF to mature GDNF are furin, PACE4, and protein convertases PC5A, PC5B, and PC7 (Lonka-Nevalaita et al. 2010). The protein convertase family is responsible for posttranslational modifications of GDNF by cleaving five consensus sites giving rise to four different peptide forms of processed GDNF. In addition, GDNF proteins with a mutation in the established furin-consensus sequence were secreted as unprocessed forms or forms with lower molecular weights compared with mature forms obtained from wild-type GDNF-overexpressing C6 cells used by Oh-hashi et al., suggesting that GDNF can be secreted with or without processing by furin-like proteases (Immonen et al. 2008; Oh-hashi et al. 2009).

Proper glycosylation is required for correct processing of GDNF protein. Piccinini et al. developed a mutant variant with an altered amino acid sequence which affected the folding of the protein. The mutant was compared with wild-type GDNF (with unchanged sequence) that was expressed in the presence of tunicamycin. Tunicamycin is an inhibitor of N-linked glycosylation, and when GDNF was expressed in its presence, the processing of the protein was similar to the mutant’s. This data demonstrates that glycosylation is necessary for proper folding and processing of GDNF protein in mammalian cells. In addition, the mutation at the glycosylation site hindered cleavage of pro-GDNF into mature GDNF (Piccinini et al. 2013).

Piccinini et al. evaluated whether the capacity of GDNF to activate its receptor complex is affected by glycosylation. The investigators showed that the unglycosylated protein, lacking the pro-sequence, is able to activate RET via GFRα1 and 2 (with less affinity). It was concluded that glycosylation is not critical for receptor activation (Piccinini et al. 2013).

Humans and rodents possess a shorter GDNF mRNA transcript. This shorter transcript has a 78 base pair deletion at the very end of GDNF exon I, which results in a 26 amino acid deletion in the pro-region of the protein. The deletion in the GDNF isoform does not affect the protein proteolytic sequence (Cristina et al. 1995; Schaar et al. 1994; Wang et al. 2008). After alternative splicing, GDNF gene encodes two mRNAs, a full-length pre-(α)pro-GDNF and a shorter pre-(β)pro-GDNF, both are cleaved to mature GDNF (Suter-Crazzorola and Unsicker 1994; Matsushita et al. 1997; Grimm et al. 1998; Penttinen et al. 2018).

Lonka-Nevalaita et al. (2010) used primary cortical neurons and the PC-6.3 cell line (neuroendocrine cells) to study intracellular processing and secretion mechanisms encoded by the (α)pro-GDNF and (β)pro-GDNF isoforms (Lonka-Nevalaita et al. 2010; Pittman et al. 1993). The research group found that, when an increase in K-Cl-induced depolarization occurs, (β)pro-GDNF and its corresponding mature GDNF secretion increased, but there was no increase of (α)pro-GDNF and its corresponding mature GDNF. In addition, after immunofluorescence analysis, it was noticed that (α)pro-GDNF and its corresponding mature GDNF were mostly localized in the Golgi complex and colocalizes with Rab3A and Rab27A (two markers for secretory granules). Furthermore, after stimulation, there is a gradual movement along the secretory pathway. In contrast, (β)pro-GDNF and its corresponding mature GDNF are mainly localized in secretogranin II and Rab3A- and Rab27A-positive vesicles, and after stimulation there is a more rapid movement along the secretory pathway (Lonka-Nevalaita et al. 2010; Wang et al. 2008).

Oh-hashi et al. established stable C6 cells that overexpressed GDNF. These cells allowed them to monitor spontaneous release of the protein, as well as its processed forms in the cells. The researchers increased GDNF secretion by stimulating the cells with high potassium and inhibited glycosylation with tunicamycin or disturbed ER-Golgi transport with brefeldin A. The wild-type GDNF-overexpressing C6 cells secreted three forms of processed GDNF. After treatment with tunicamycin, GDNF secreted with the highest molecular weight showed the same mobility on electrophoresis as recombinant human GDNF missing a whole pro-domain (Oh-hashi et al. 2009). When mutations occurred in the pro-domain and two cysteines located at the C-terminal of GDNF protein, secretion to the culture medium was significantly decreased. This data suggests that pro-domains and C-terminal cysteines of GDNF have a critical role in its processing and secretion in cultured astrocytes and C6 cells (Oh-hashi et al. 2009).

An important aspect of GDNF internalization is the encapsulation of the neurotrophic signal by endocytosis of both receptor and ligand. RET receptor when activated by GDNF has been uncovered to associate with AP2 and clathrin at the plasma membrane and possibly facilitating clathrin-mediated endocytosis similarly to NGF (Beattie et al. 2000; Crupi et al. 2015; Howe et al. 2001). Further studies are required to understand long-distance trafficking of GDNF.

SorLA is a member of the sortilin-related receptor family that is unified by the vacuolar protein sorting protein 10p domain (Jacobsen et al. 1996; Glerup et al. 2013; Willnow et al. 2008). SorLA possesses a cytoplasmic tail with a number of consensus binding sites for adaptor proteins that aid internalization from the cell surface, Golgi-endosome transport, and retrograde sorting to the trans-Golgi network (Nielsen et al. 2007). Glerup et al. (2013) studied the mediating interactions of SorLA as a sorting receptor for the GDNF/GFRα1 complex, leading the complex from the cell surface to endosomes. GDNF subsequently is targeted by lysosomes, and GFRα1 is recycled, thus making an effective clearance pathway. Furthermore, the SorLA/GFRα1 complex targets RET for endocytosis but not for degradation, which affect neurotrophic activities caused by GDNF like the survival of primary dopaminergic neurons (Glerup et al. 2013). Further studies are needed to understand GDNF internalization and trafficking.

## GDNF signaling

The RET receptor occurs in the central and peripheral nervous system during development (Sanicola et al. 1997). GDNF and the rest of the GLFs use RET as a signaling receptor, but RET can only be activated in the presence of a co-receptor glycosylphosphatidylinositol-linked GDNF (GFRα). As previously reviewed by Sariola and Saarma, ligand-binding specificity for the GFLs is dependent on the GFRα receptor proteins. There are a total of four GFRα proteins that interact with the GFLs: GFRα1, GFRα2, GFRα3, and GFRα4. GDNF preferably binds to GFRα1, but studies using GFRα1 knockout mice suggest that GDNF can act via GFRα2 with less affinity (Cacalano et al. 1998; Sariola and Saarma 2003; Schuchardt et al. 1994; Jing et al. 1997; Enomoto et al. 1998).

**Fig. 1:**
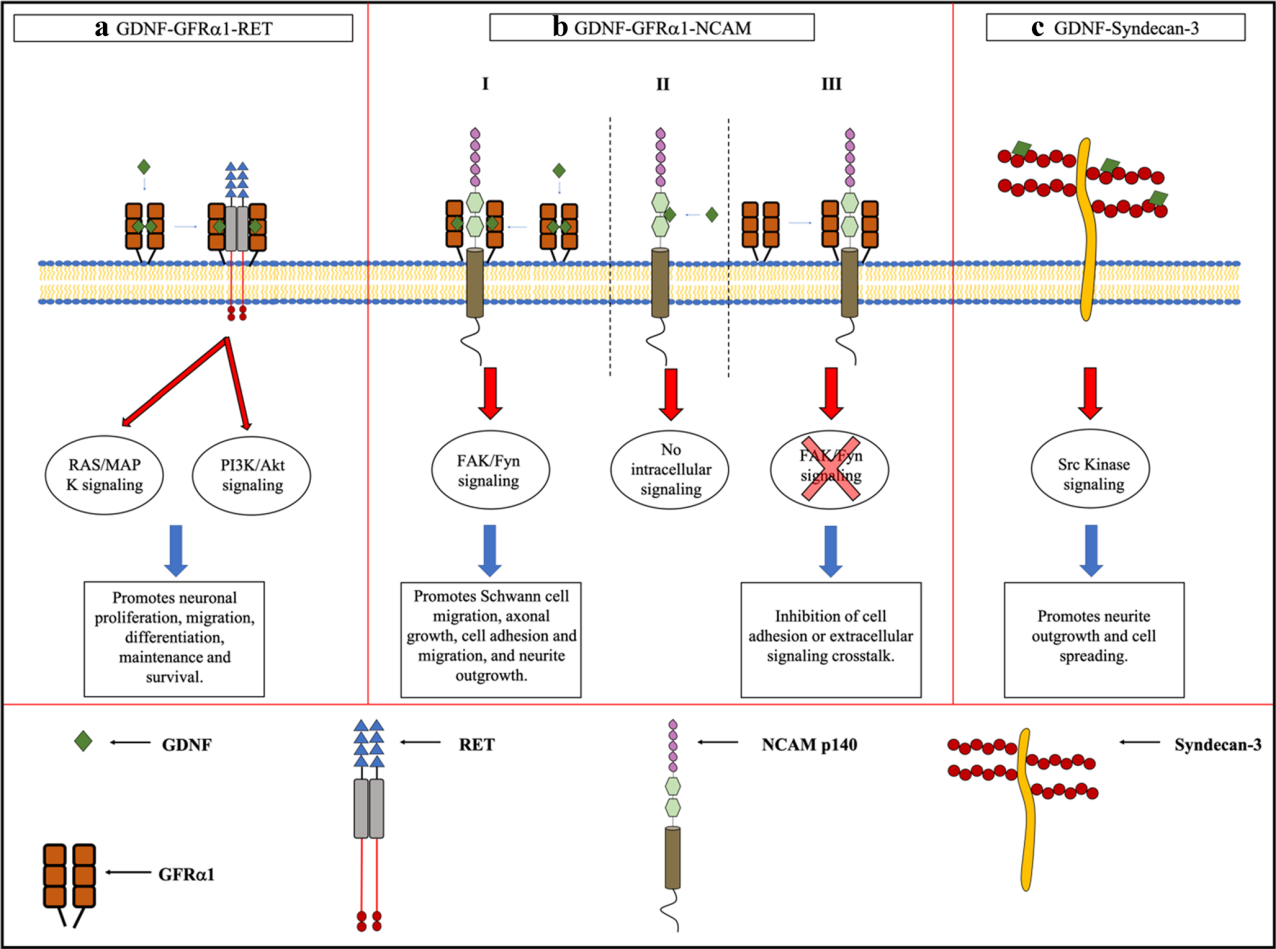
GDNF and its receptors. **A** GDNF-GFRα1-RET receptor signaling. A functional GDNF dimer associates with a GPI-anchored GFRα1 receptor. The GDNF-GFRα complex links with a RET tyrosine kinase receptor. The GDNF-GFRα1-RET complex triggers intracellular signaling pathways that promote neuronal survival. **B** GDNF-GFRα1-NCAM receptor signaling. **B-I** GDNF binds to a GFRα1 receptor. The GDNF-GFRα1 complex associates with the NCAMp140 isoform and triggers intracellular signaling. **B-II** GDNF or another GFL binds directly to the NCAM receptor; no intracellular signaling is triggered. **B-III** GFRα1 binds directly with NCAM without GDNF, and it causes cell adhesion inhibition. **C** Matrix-bound GDNF-syndecan-3 signaling. When GDNF is immobile and matrix bound, it can be associated with a transmembrane heparan sulfate (HS) proteoglycan called syndecan-3. This binding can promote neurite outgrowth and cell spreading

GDNF is functional when it is a homodimer stabilized by a disulfide bond. GDNF first binds to a lipid raft-resident glycosylphosphatidylinositol (GPI)–anchored GFRα1 receptor (preferentially) and forms a high-affinity complex as shown in Fig. 1A. This complex brings two RET molecules causing transphosphorylation of specific tyrosine residues in their domains and intracellular signaling (Butte 2001; Jing et al. 1996; Stromberg et al. 1993; Trupp et al. 1995; Sariola and Saarma 2003; Airaksinen and Saarma 2002). The activation of RET triggers the mitogen-activated protein kinase (MAPK), phosphoinositositide-3-kinase (PI-3K), Erk, and Akt pathways which are attributed to act in promotion of cell survival (Airaksinen and Saarma 2002; Sariola and Saarma 2003; Kim and Kim 2018).

Membrane microdomains are enriched with signaling proteins like the Src family kinases (SFK). This enrichment has led to the reasoning that these microdomains could function as a specialized signaling organelle (Anderson 1998). Work from Tansey et al. (2000) showed that, to obtain maximal levels of GDNF-mediated bioactivity and efficient downstream signaling, RET has to be recruited to lipid rafts by GFRα1 receptors and interact with SFKs found in these microdomains. In addition, SFKs only interact with RET when the receptor is located on the membrane microdomains (Tansey et al. 2000). Encinas et al. (2001) studied the RET-SFK association in downstream signaling caused by GDNF. The researchers found SFK activity, in particular p60Src, was needed for best GDNF-mediated Akt and MAPK phosphorylation. In addition, Src promoted neuronal survival through PI-3K pathway activity. When a PI-3K inhibitor is used, it prevented GFL-mediated and Src-mediated neuronal survival, but it had no effect on NGF-mediated survival, suggesting Src was particular for the GFL-mediated neuronal survival. Taking this data together, the researchers suggest Src as major signaling molecule for GDNF-mediated bioactivity (Encinas et al. 2001).

GDNF signaling can occur without the presence of RET. GFRα is more widely expressed in the nervous system compared with RET (Trupp et al. 1997; Yu et al. 1998). That being the case, GFRα receptors should have an alternate way to signal, independent from RET, probably with novel transmembrane proteins (Poteryaev et al. 1999; Trupp et al. 1999). Neural cell adhesion molecule (NCAM) is an adhesion molecule found in the nervous system and in the skeletal muscle, and it is involved in cell migration, synaptic plasticity, and neurite outgrowth during development (Crossin and Krushel 2000; Ronn et al. 2000; Schachner 1997). Paratcha et al. studied RET-independent signaling by GDNF in both glial and neuronal cells. They found a compelling similitude with intracellular pathways that were activated by the p140^NCAM^ NCAM isoform. The researchers demonstrated that the NCAM isoform interacts directly with GDNF family ligands along with GFRα receptors and mediates GDNF signaling with no RET presence. GDNF acting via the NCAM pathway promotes Schwann cell migration and axonal growth in cortical and hippocampal neurons (Paratcha et al. 2003). GFLs, when GFRα receptors are present, can bind with p140^NCAM^ and activate Src-like kinase Fyn and focal adhesion kinase FAK, a non-receptor tyrosine kinase that plays a role in cytoskeletal rearrangement as shown in Fig. 1B-I. GFLs can also bind directly to the NCAM isoform, but with much less affinity and does not trigger any intracellular signaling as seen in Fig. 1B-II. When GDNF is not present and GFRα1 receptor interacts with NCAM, it inhibits NCAM-mediated cell adhesion (Fig. 1B-III), suggesting potential difference in physiological effects, but further studies are required (Beggs et al. 1997; Paratcha et al. 2003; Saarma 2009; Kim and Kim 2018).

Pozas and Ibáñez (2005) demonstrated that GDNF has the ability to promote differentiation and migration of embryonic cortical GABAergic neurons that lack both RET and NCAM, suggesting another receptor must exist mediating GDNF-dependent processes in cortical development (Pozas and Ibáñez 2005; Bespalov et al. 2011). Bespalov et al. (2011) showed that GFLs (with the exception of persephin) can use a different receptor. When GFLs are immobilized and matrix bound, they can associate with a transmembrane heparan sulfate (HS) proteoglycan called syndecan-3 (Fig. 1C). The researchers found GFL-syndecan-3 binding mediates neurite outgrowth and cell spreading with the collaboration of Src kinase activation. They discovered that GDNF-syndecan-3 promotes migration of cortical neurons, and when mice lack either one of those components, they have a reduced number of GABAergic neurons (Bespalov et al. 2011). Altogether, these studies suggest that GDNF may act through various pathways, mediating growth, differentiation, and migration of neurons. Once GDNF binds to one of its receptors, it must be incorporated and transported.

## Retrograde transport in motor neurons

Following the neurotrophic factor theory, the target cell releases a trophic “substance.” This trophic substance is available in limited supply at the time of natural apoptosis during development. The trophic factor released from the target cell reaches an axon terminal, and it is shipped in a retrograde form to the cell body of the innervating neuron to promote survival (Oppenheim 1989). Retrograde spreading of a neurotrophic signal is performed by cellular internalization of the neurotrophic factor along with its receptor at the distal location. After internalization, compartmentalization into signaling endosomes occurs, followed by motor protein-based transport toward the cell body (Wu et al. 2009; Zahavi et al. 2015; Zahavi et al. 2017). Researchers have showed both retrograde and anterograde transport of GDNF (Haase et al. 2002; Leitner et al. 1999; Rind and von Bartheld 2002; Russell et al. 2000; Zahavi et al. 2015) For the purpose of this review, we will discuss retrograde transport of GDNF in MNs. Further studies are needed to better understand the physiological effects of anterograde transport in MNs.

Expression of GDNF in skeletal muscle suggests the existence of retrograde transport and signaling from target tissue, even at adult ages (Henderson et al. 1994; Nguyen et al. 1998). GDNF is considered the first MN-specific neurotrophic factor discovered (Treanor et al. 1996). Leitner et al. (1999) studied retrograde transport of some of the members of the GDNF family in vivo. The researchers performed sciatic injections with radiolabeled GDNF and neurturin and then used autoradiography to determine retrograde transport in MNs in the lumbar region of the spinal cord in adult Sprague Dawley rats. Examination of the spinal cord from the injected rats showed more radiolabeled GDNF localized to ventral MNs compared with neurturin. This result shows retrograde activity from GDNF and physiological differences in the actions of the GFLs (Leitner et al. 1999).

Haase et al. (2002) studied MNs that express the erythroblast transformation-specific (ETS) transcription factor Pea3 in a mouse model. These MNs project their axons toward the cutaneous maximus muscle and latissimus dorsi muscle, which show expression of GDNF. The researchers noticed GDNF when lost in skeletal muscle impaired motor nerve projection and downregulation of Pea3 in MNs. Due to these findings, it is suggested that retrograde transport of GDNF signaling is necessary for axon elongation and expression of Pea3 in neuronal cell bodies. In addition, the axon-promoting effects by GDNF are not merely a local event occurring in target skeletal muscle (Haase et al. 2002; Ito and Enomoto 2016).

More recent research from Zahavi et al. (2015) developed an in vitro microfluidic platform that contained MN somata on one side and muscle cells on the other. The cell body of the MN and muscle cells were connected by motor axons that extended through microgrooves to form neuromuscular junctions (NMJs) that were functional. The benefit of having this in vitro compartmentalized system separating cell bodies from their axons and target cells provides the opportunity to study local versus distal signals and monitor retrograde and anterograde transport (Taylor et al. 2005; Zahavi et al. 2015). By using this model, Zahavi and colleagues managed to characterize spatial specificity of the effects of GDNF. They noticed that when GDNF is added at the soma survival pathway signaling via AKT is activated. In contrast, when GDNF is applied at the muscle compartment side, it promoted axonal growth at the axon tips and innervation of muscle cells. In addition, the researchers were able to visualize trafficking of GDNF in a retrograde fashion, from muscle cell to neuron (Zahavi et al. 2015).

## Muscle-derived GDNF effects on innervation maintenance

GDNF plays a role in maintaining the NMJ. When insufficient levels of GDNF are present, neuronal cell death can occur, resulting in compromised structure and function of NMJs. Springer et al. (1995) reverse-transcribed the total RNA from adult rat skeletal muscle and amplified it for GDNF mRNA using PCR. They found two forms of GDNF and noticed that one of the transcripts (GDNF633) was upregulated in denervated rat skeletal muscle following 1–2 weeks of an axotomy procedure. In a similar fashion, Zhao et al. (2004) studied the expression of GDNF mRNA present in four populations of skeletal muscle: healthy skeletal muscle, denervated muscle, denervated muscle receiving sensory input, and denervated muscle in which innervation was immediately repaired. The researchers concluded that denervated muscle had the greatest GDNF expression, followed by levels in the muscle that received sensory protection, and the muscle that underwent immediate repair. The healthy skeletal muscle displayed the lowest levels of GDNF mRNA, which provides strong evidence for increased expression of GDNF during reinnervation of the NMJ (Zhao et al. 2004). These results provided further indications of the roles of muscle innervation in determining the expression profile of alternatively spliced forms of GDNF, GDNF working as a target-derived neurotrophic factor for neurons that innervate skeletal muscle, and the effects of providing some sort of therapy on modulating GDNF synthesis (Springer et al. 1995; Zhao et al. 2004).

Duchenne muscular dystrophy (DMD) is a genetic disorder that causes alterations in dystrophin leading to muscle degeneration and frailty. A study done by Lie and Weis (1998) examined the effects of denervation in human muscle on GDNF upregulation. The researchers compared the expression of GDNF transcripts in healthy muscle, denervated muscle, and muscle biopsies from patients diagnosed with DMD. The researchers determined that GDNF expression was significantly higher in muscle that was denervated when compared with normal or DMD-affected muscle (Lie and Weis 1998). A possible reason for this result is that in DMD patients there is still partial innervation and there is less pressure in trying to maintain nerve to muscle contact when compared with denervated muscle. It is significant to note that the work done by Lie and Weis (1998) showed that GDNF mRNA expression in humans following denervation follows a similar pattern like in rats (Lie and Weis 1998; Springer et al. 1995; Zhao et al. 2004). In addition, greater levels of GDNF were expressed in cases where a patient had a rapidly progressive neurogenic atrophy. GDNF expression in patients that had DMD was not significantly different compared with that in the controls. Altogether, Lie and Weis (1998) demonstrated evidence of the role of denervation and GDNF expression, but not dystrophy. We recommend that further human studies are needed to provide more insight on the potential of using GDNF as a re-innervating and regeneration therapy.

Nguyen et al. generated a mouse model where muscle fibers make excess quantities of GDNF. The researchers noticed GDNF overexpression caused hyperinnervation at the motor end plates found in muscle fibers. GDNF made in muscle could be acting as a synaptotrophin at the NMJ. Interestingly enough, other neurotrophic factors like NT-3 and NT-4, when overexpressed, did not provoke hyperinnervation of muscle fibers (Nguyen et al. 1998).

## Exercise and GDNF expression

It has been suggested that exercise is a potential approach to enhance functional recovery of central and peripheral nerve injuries, delays neurodegenerative diseases, and has a potential for regulating neurotrophic factor signaling as reviewed by Cobianchi et al. (2017). It is still not completely understood how physical activity could increase neurotrophic factor gene expression in active muscle. Wehrwein et al. (2002) explored the changes in neuromuscular activity on GDNF content in rat skeletal muscle. Rats followed 4 weeks of walk training on a treadmill, and it was noticed that GDNF protein content increased in the pectoralis major, soleus, and gastrocnemius muscle. In addition, hindlimb unloading for 2 weeks was explored, and it was noticed that GDNF protein content decreased in the soleus and gastrocnemius muscles but increased in the pectoralis major suggesting that activity-dependent regulation of GDNF occurs in rat skeletal muscle (Wehrwein et al. 2002).

McCullough et al. (2013) explored changes in GDNF protein levels in the spinal cord following exercise. The researchers demonstrated that 2 weeks of either swimming or running altered GDNF protein content levels in the lumbar spinal tissue of both young and old rats. The L1–L3 lumbar region of the spinal cord was obtained from sedentary control and exercised 6-month animals and 24-month-old animals. The spinal tissue was processed for GDNF protein content for ELISA and Western blot assays. Results showed that GDNF protein content significantly increased in lumbar spinal tissue from exercised animals compared with that from sedentary age-matched animals (McCullough et al. 2013). In addition, lumbar spinal cord sections were immunolabeled with anti-choline acetyltransferase (ChAT) for somatic MNs (Wetts and Vaughn 1996) and with anti-GDNF. It was noticed by McCullough et al. (2013) that MN cell body size and vesicle-like structures of GDNF increased in spinal tissue from the exercised rats compared with their sedentary counterpart (McCullough et al. 2013).

Using a similar exercise regimen, Gyorkos et al. (2014) discovered that 2 weeks of running and swimming training can promote changes in GDNF expression and NMJ structure in both slow- and fast-type muscles. GDNF protein content was measured using ELISA. There was a significant increase of GDNF protein content level in the soleus muscle, while the extensor digitorum longus (EDL) trended toward an increase in GDNF protein content (Gyorkos et al. 2014). The morphology of the NMJ was analyzed by using α-bungarotoxin, which binds to nicotinic acetylcholine receptors, thus labeling the post-synaptic end plates. The total area (μm^2^) of post-synaptic end plates significantly increased in the soleus muscle following swimming exercise compared with sedentary control animals. In contrast, the total area of end plates significantly decreased in the EDL muscle after running training, and no differences were noticed after swimming training (Gyorkos et al. 2014). Taken together, the works of McCullough et al. (2013) and Gyorkos et al. (2014) demonstrate that physical exercise is sufficient to increase GDNF protein content in the spinal cord, within skeletal muscles, and at the NMJ. In addition, it offers new insights for developing exercise regimens as a form of additional therapy and/or treatment for patients with MN disease.

## GDNF as a potential treatment for motor neuron diseases

Amyotrophic lateral sclerosis (ALS) is a disease that mainly affects MNs causing loss of voluntary movement. Due to the potential neuroprotective effects of GDNF, many studies have focused on ameliorating neurodegenerative diseases by adding exogenous GDNF. Researchers used human mesenchymal stem cells to deliver GDNF directly into skeletal muscle of familial ALS model rats. The rats showed increased muscular levels of GDNF, retrograde transport of GDNF protein into motor neurons, neuroprotective effects on neuron survival, and function at the neuromuscular junction (Suzuki et al. 2008).

A similar neuroprotective effect was observed in transgenic ALS mice following intramuscular injection of an adeno-associated virus (AAV) carrying the gene for GDNF (Wang et al. 2002). Wang et al. (2002) noticed that the addition of AAV-GDNF led to longer expression of transgenic GDNF in myofibers, mostly concentrated at the NMJ. In addition, the transgenic GDNF prevented motor neuron death and protected axons that innervate skeletal muscle and muscle treated with the AAV-GDNF showed atrophy inhibition (Wang et al. 2002). These studies suggest that cellular incorporation of GDNF may help prevent the neuromuscular damage associated with ALS.

Thomsen et al. used human cortical-derived neural progenitor cells modified to secrete GDNF (hNPC^GDNF^) which were transplanted to the cortex of an ALS rat model (SOD1^G93A^). This study is the first to show the transplanted cells survived, eventually migrated, developed into mature astrocytes, and secreted GDNF protein. Results from this study showed MN protection, pathology of the disease was slowed down, and the animals experienced an increased life span. These same cells were also used in the cortex of a primate model (*cynomolgus macaques*) and also had noticeable GDNF expression, and negative effects relating to behavior in a period over 30 days were not observed (Thomsen et al. 2018). This study shows promise in using hNPC^GDNF^ as possible treatment against MN disease. In addition, the successful use of these cells in a nonhuman primate facilitates and opens the door to further explore the hNPC^GDNF^ and potentially use them in clinical trials for humans.

An unfortunate effect of developing an embryonic GDNF knockout mice is renal agenesis causing neonatal death (Moore et al. 1996; Sanchez et al. 1996). Studies using conditional GDNF knockouts have been developed, but none have completed total gene removal as reviewed by Duarte Azevedo et al., which has led to argumentative conclusions (Duarte Azevedo et al. 2020). Further studies are needed to fully elucidate the physiological functions of GDNF.

## Conclusion

Research regarding GDNF demonstrated a variety of neuroprotective roles for mammalian neurons and, as discussed in this review, GDNF acts as a potent neurotrophic factor for MNs. Through the research performed in the past few decades, GDNF has been found to aid in establishing new synapses between MN and target tissues, promoting growth, maintenance, and survival of neurons. With advancements in research and medicine, researchers can become more adept at delivering GDNF and other neurotrophic factors as promising candidates for a new way of therapy, preventing or treating neuro-related conditions. Nevertheless, there remains a significant gap in the knowledge of the pathways triggered by the complex of GDNF and GFRα following its activation of RET. Hopefully, future research will address the downstream effects of this intracellular signaling pathway and other pathways which will provide a stronger relationship between GDNF and its neuroprotective properties within MNs. Enhancing our understanding of GDNF and its signaling pathways will allow for the discovery of possible therapeutic approaches for applications of a broad range of neurological disorders such as ALS. Moreover, studying active-dependent GDNF release from skeletal muscle can aid in better creating exercise regimens as additional treatment for maintenance of the neuromuscular system.
